# Dynamics of Stride Interval Characteristics during Continuous Stairmill Climbing

**DOI:** 10.3389/fphys.2017.00609

**Published:** 2017-08-23

**Authors:** Peter C. Raffalt, Srikant Vallabhajosula, Jessica J. Renz, Mukul Mukherjee, Nicholas Stergiou

**Affiliations:** ^1^Julius Wolff Institute for Biomechanics and Musculoskeletal Regeneration, Charité–Universitätsmedizin Berlin Berlin, Germany; ^2^Department of Biomedical Sciences, University of Copenhagen Copenhagen, Denmark; ^3^Department of Physical Therapy Education, School of Health Sciences, Elon University Elon, NC, United States; ^4^Department of Biomechanics and Center for Research in Human Movement Variability, University of Nebraska Omaha Omaha, NE, United States; ^5^Department of Environmental Agricultural and Occupational Health, College of Public Health, University of Nebraska Medical Center Omaha, NE, United States

**Keywords:** temporal structure of variability, detrended fluctuation analysis, entropy, stair biomechanics, stride-to-stride fluctuations

## Abstract

It has been shown that statistical persistence in stride intervals characteristics exist during walking, running and cycling and were speed-dependent among healthy young adults. The purpose of this study was to determine if such statistical persistence in stride time interval, stride length and stride speed also exists during self-paced continuous stairmill climbing and if the strength is dependent on stepping rate. Stride time, stride length, and stride speed were collected from nine healthy participants during 3 min of stairmill climbing at 100, 110, and 120% of their preferred stepping rate (PSR) and 5 min of treadmill walking at preferred walking speed (PWS). The amount of variability (assessed by standard deviation and coefficient of variation) and dynamics (assessed by detrended fluctuation analysis and sample entropy) of the stride time, stride length, and stride speed time series were investigated. The amounts of variability were significantly higher during stairmill climbing for the stride time, stride length, and stride speed and did only change with increased stepping rate for stride speed. In addition to a more irregular pattern during stairmill climbing, the detrended fluctuation analysis (DFA) revealed that the stride length fluctuations were statistical anti-persistent for all subjects. On a group level both stride time and stride speed fluctuations were characterized by an uncorrelated pattern which was more irregular compared to that during treadmill walking. However, large inter-participant differences were observed for these two variables. In addition, the dynamics did not change with increase in stepping rate.

## Introduction

Walking and running are activities of daily living that are performed on a regular basis. The repetitive cyclic motion of the lower extremities during these activities suggests that the spatio-temporal characteristics of such tasks are predictable with low variability. Previous researchers have quantified this variability using measurements that computed both the amount and temporal structure of variability. It has been shown that during walking and running, healthy adults show a lesser amount of variability, often measured using standard deviation and coefficient of variation parameters as compared to adults with neurological diseases like Parkinson's disease (Blin et al., 1991; Hausdorff et al., 1996, 1998).

Regarding the temporal structure of variability, the dynamics of walking have been quantified by examining if statistical persistence were present in spatio-temporal parameters in strides at different time points (i.e., earlier and later strides). Examining these stride-to-stride fluctuations can help us understand the relationship between two strides at different time points (Hausdorff, 2007; Jordan et al., 2007). Healthy young adults exhibit statistical persistence in their stride interval while walking and running (Hausdorff et al., 1996, 1997; Jordan et al., 2006; Dingwell et al., 2010; Terrier and Deriaz, 2012; Marmelat et al., 2014; Terrier, 2016). However, these temporal dynamics have been shown to change to statistical anti-persistence under the influence of auditory and visual metronomic cues (Terrier and Deriaz, 2012; Marmelat et al., 2014; Terrier, 2016). Moreover, Hausdorff and colleagues showed that with aging and diseases, such as Huntington's disease, there is a loss of persistence in the stride-to-stride intervals during walking (Hausdorff et al., 1997). While stride length exhibits statistical persistence similar to that of stride time intervals, stride speed exhibits anti-persistence (Dingwell and Cusumano, 2010; Dingwell et al., 2010; Terrier and Deriaz, 2012; Terrier, 2016). The dynamics of stride length are equally affected by auditory and visual cues and changed to exhibit anti-persistence (Terrier and Deriaz, 2012; Terrier, 2016).

The literature appears to be inconclusive regarding the effects of speed on the statistical persistence during walking and running. A U-shaped relationship between speed and the statistical persistence during walking (Jordan et al., 2007; Jordan and Newell, 2008) and running (Jordan et al., 2006) has been reported with an increase in the strength of the persistence at speeds below and above the preferred walking or running speed. In contrast, other studies have observed no speed effect on the statistical persistence during walking (Dingwell and Cusumano, 2010; Terrier and Deriaz, 2012) and running (Lindsay et al., 2014). The former observations have been interpreted as a higher adaptability of the system at the preferred speed than at higher and lower speeds. It has previously been hypothesized that the statistical persistence is stronger at slower and faster speeds because of the “biological stress” on the central nervous system (West and Scafetta, 2003; Jordan et al., 2006). In their simulation model, West and Scafetta (2003) suggested that change in walking speed was a stress mechanism of internal origin that would increase the strength of the statistical persistence and synchronization with a metronome was a stress mechanism of external origin that would decrease the strength of the statistical persistence. Thus, walking in high speed conditions is accomplished by a range of combinations of short stride times and long stride lengths, while, the opposite is true for walking in low speed conditions. In contrast, maintaining a constant intermediate walking speed could be accomplished by a wider range of combinations of short, intermediate, and long stride length paired with short, intermediate, and long stride times. An activity like continuous stairmill climbing which is a common yet challenging task, could to be solved through a relatively narrow range of combinations of stride length (basically fixed) and stride time. Thus, it could be hypothesized that similar to walking at higher or lower speeds, continuous stairmill climbing potentially constitutes a greater biological stress on the motor control system compared to walking.

Previous studies using joint kinematics and kinetics have shown that stair-climbing places greater demands compared to overground walking (Reeves et al., 2009; Samuel et al., 2011; Vallabhajosula et al., 2012a,b). However, it is unknown if the dynamics of stride-to-stride time intervals exhibits statistical persistence during a challenging task like continuous stairmill climbing. Further it is also unknown if the strength of the statistical persistence is speed-dependent. Though continuous stair-climbing also involves repetitive cyclical motion of the lower extremities like walking, running and cycling, there is a greater risk of experiencing a fall while trying to clear the intermediate step. In turn, this could affect the stepping pattern and the stride-to-stride fluctuations.

The purpose of this exploratory study was 2-fold. First, we wanted to determine the existence of statistical persistence in stride time interval, stride length, and stride speed during self-paced continuous stair-climbing. Given the demanding nature of the task, we hypothesized that in comparison to walking, the strength of statistical persistence during continuous stair-climbing would be increased. Second, we determined if the strength of the statistical persistence was dependent on stairmill climbing speed. We hypothesized that continuous stairmill climbing at greater speeds would result in an increase in the strength of the statistical persistence than stairmill climbing at the preferred speed. This will be due to the additional demand on the motor control system to ensure a faster yet safer continuous stair-climbing. To strengthen the outcome of this study and to enhance the interpretation of the proposed hypotheses, we also investigated the regularity in stride time interval, stride length, and stride speed time series (Pincus, 1991, 1995; Pincus and Goldberger, 1994). Entropy measures (approximate and sample entropy) have previously been used to determine physiological changes with aging (e.g., increased irregularity in heart beat time intervals have been observed in older adults Kaplan et al., 1991). In the present study sample entropy was used to establish if stair-climbing induces a more periodic or random structure in the stride-to stride fluctuation compared to walking.

## Materials and methods

### Participants

Nine healthy participants (5 females and 4 males; mean ± standard deviation: age, 25.2 ± 4.9 years; height, 1.71 ± 0.10 m; mass, 69.1 ± 13.8 kg) were included in the current study. Inclusion criteria were: (1) adults aged between 19 and 35 years; (2) must be familiar and able to use the stairmill and treadmill devices and (3) able to provide informed consent. Exclusion criteria were: known sensory, neuromuscular, skeletal or cardiovascular disorders that may affect a person's gait pattern, or inability to use the study's stairmill without the use of the handrail. All participants were informed of the experimental conditions and gave their informed written consent to participate in the study and to the publication of identifying images. The study was approved by the Institutional Review Board of the University of Nebraska Medical Center, and the study was carried out in accordance with the approved guidelines.

### Instrumentation

**Figure 1:**
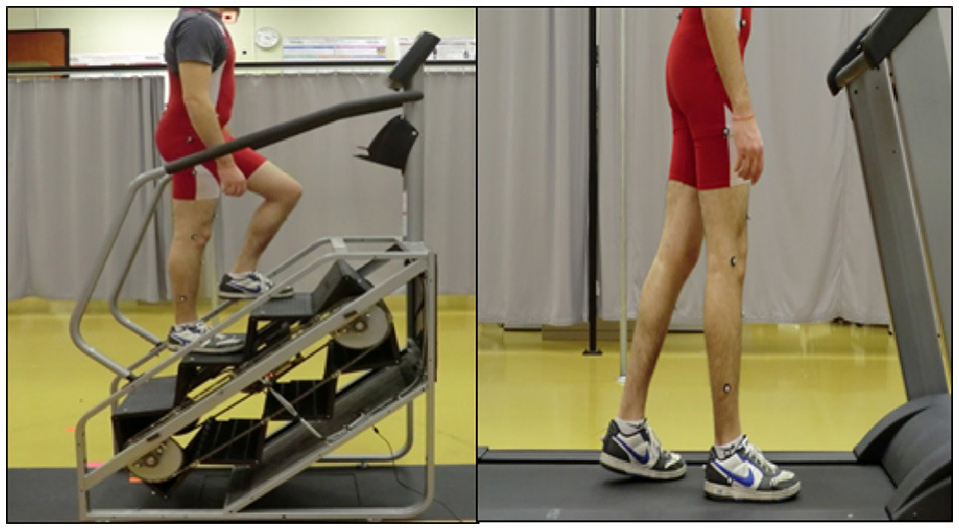
Pictures showing experimental set-up with the participant walking on stairmill **(left)** and treadmill **(right)**.

The SC916 motorized stairmill (StairMaster, Fitness Direct, San Diego, CA) was used in this study to allow the participants to perform continuous stair-climbing and a T280S flat motorized treadmill was used for the treadmill walking trial (Bodyguard Fitness, QC, Canada). Kinematics data were collected at 60 Hz using a 12 high-speed camera system (Motion Analysis Corp., Santa Rosa, CA) from a retro-reflective marker placed on the head of the 2nd meta-tarsal of the participant's right leg. The experimental setup is shown in Figure 1. Markers were also placed on each step of the stairmill and on the treadmill.

### Procedures and protocol

Before collecting the data, participants selected their preferred stepping rate (PSR) to perform continuous stair-climbing without using handrails. The PSR was confirmed by increasing the stepping rate until the participant reported discomfort with further increase. Then the stepping rate was decreased until the participant asked to increase the stepping rate. This was repeated until the participant reported a comfortable stepping rate which was used as a PSR. The participants were blinded from the numerical value of the stepping rate that was displayed on the stairmill. Each participant was given 1 min to familiarize themselves stepping at their PSR. Data collection began after at least 2 min of rest post-familiarization. Data were collected in three conditions for 3 min each: PSR, fast stepping rate (110%PSR) and very fast stepping rate (120%PSR). The order of the conditions was randomized. A resting period of at least 3 min was provided between each testing condition. After collecting data on the stairmill and sufficient rest, the preferred walking speed (PWS) on the treadmill was determined by repeatedly increasing and decreasing the walking speed above and below the speed that the participant reported as comfortable. Following another rest period, participants performed a 5 min walking trial at PWS, which in average was 1.19 (0.24) m/s.

### Data analysis

The right leg stride time, right leg stride length and right leg stride speed were calculated from raw marker position using a custom-written script in Matlab (Mathworks, Inc., Natick, MA). Right leg stride time was defined as the time between two consecutive toe-off events with the right leg. Right leg stride length was defined as the distance traveled by the treadmill and the stairmill during the corresponding right leg stride time. The toe-off event was determined using the position of the marker placed on the head of the 2nd metatarsal. The toe-off event was defined as the time point when the vertical position of the marker on the 2nd meta-tarsal increased rapidly at the end of the contact phase. All the participants had at least 65 strides in the PSR, 110%PSR and 120%PSR stairmill conditions and at least 167 strides during treadmill walking. Thus, 65 strides for all stairmill trials and 167 strides from all treadmill walking trials were extracted for further analysis. The mean, standard deviation, and coefficient of variation of the right stride time, stride length and stride speed were computed under all the stairmill and treadmill conditions, to evaluate amount of variability in the time series. Additionally, the present study applied three methods to evaluate different aspects of the dynamics of the investigated time series.

The presence of statistical persistence or anti-persistence of the right stride time intervals and stride length were investigated using the Detrended Fluctuation Analysis (DFA) technique. DFA is a statistical method to quantify the complexity of a physiological signal. It has the ability to detect the presence of statistical persistence in the data series. These correlations are part of multifractal cascades that exist over a wide range of time scales (Hausdorff et al., 1996). It is known that many biological signals are noisy, heterogeneous, and also exhibit non-stationary characteristics that can affect the specific correlation properties of the signal. However, one of the primary advantages of using the DFA method is that it allows for the detection of statistical persistence within noisy signals with embedded polynomial trends that can match the true correlation in the fluctuation of the signal (Peng et al., 1995; Chen et al., 2002).

The DFA algorithm was implemented by using custom-script written in Matlab based on the methods used by Peng et al. (1995). The DFA method begins by first forming an accumulated sum of the time series and sections it into windows. Then the log of the average size of fluctuation for a specific window size is plotted against the log of the window size. Briefly, Equation 1 listed below shows that if B(i) is the ith interval and Bave is the average interval then:

(1) $$\begin{array}{r} y\left(k\right)=\overset{k}{\underset{i}{\sum }}\left[B\left(i\right)-B_{ave}\right] \end{array}$$

The time series is then divided into boxes of equal length, n. In each of length n, a least square line is fit to the data. The y coordinate of the straight-line segments is designated by *y*_*n*_(*k*). The time series is detrended, *y*_*n*_(*k*), by subtracting the local trend, *y*_*n*_(*k*), in each box and then the root mean square fluctuation of this integrated and detrended time series is calculated by the Equation 2 listed below. This same calculation is then repeated across the entire time series to provide a relationship between F(n), which is the average fluctuation as a function of box size, and the box size n. A linear relationship on a double log graph indicates the presence of scaling. The fluctuations can be characterized by the scaling exponent α, which is determined by finding the slope of the line relating log*F*(*n*) to log*n* (Peng et al., 1995).

(2) $$\begin{array}{r} F\left(n\right)=\;\sqrt{\frac{1}{N}\overset{N}{\underset{k=1}{\sum }}{\left[y\left(k\right)-y_{n}\left(k\right)\right]}^{2}} \end{array}$$

If the alpha-value calculated by DFA is > 0.5 then there is a presence of statistical persistence, meaning that longer stride interval in the past are likely to be followed by another longer stride interval. If the alpha-value is < 0.5 then there is a presence of anti-persistence, meaning that longer stride interval in the past are likely to be followed by a shorter stride interval. An alpha-value of 0.5 indicates an uncorrelated white noise like pattern of the time series (Hausdorff et al., 1996, 1997; Hausdorff, 2007). In the current study, a box size range of [2, N] where *N* = 65 was used for DFA for stairmill data and *N* = 167 for treadmill walking with the box sizes logarithmically spaced (Jordan et al., 2006). The scaling region was in all cases set to 1:14. A validation of the chosen box size is presented in the Supplementary Material 1. Since the used data series were relatively short for DFA, additional analyses were performed to ensure that the length of the data series did not affect the reliability and precision. These analyses can be found in Supplementary Material 2.

Furthermore, a secondary measure of the temporal structure of variability was calculated to enhance our interpretation of the DFA results. The sample entropy of the stride-to-stride time fluctuation was calculated by a custom written matlab script based on the algorithm by Richman and Moorman (2000) (Equation 3). Sample entropy was defined as the negative logarithm for conditional properties that a series of data points within a certain distance, m, would be repeated within the distance m+1 (Richman and Moorman, 2000).

(3) $$\begin{array}{r} SaEn\left(m,r,N\right)=-\ln\;\left[\frac{A^{m+1}\left(r\right)}{B^{m}\left(r\right)}\right] \end{array}$$

Where A is the number of similar vector length, m+1, which falls within a relative tolerance limit (r times standard deviation of the time series) and B is the number of similar vector length, m, which falls within the tolerance limit (Yentes et al., 2013).

To control for parameter consistency of the selected vector length m and tolerance limit r, sample entropy was calculated using m of 2 and 3 and r of 0.1, 0.15, 0.2, 0.25, and 0.3 (Yentes et al., 2013). After inspection of the sample entropy consistency, m of 2 and r of 0.2 were used for the presented results.

To investigate the nature of the fluctuation in the time series a surrogate analysis was applied as demonstrated by Theiler et al. (1992). The applied surrogate procedure includes a test of the null hypothesis that the fluctuation in the time series could be generated by a linearly autocorrelated Gaussian process. Firstly, 39 surrogate data sets were generated from the stride-time and stride length series from each stairmill and treadmill condition and from each participant using the method presented by Theiler et al. (1992) (algorithm 1). This algorithm was chosen to ensure that the surrogate time series would contain a stochastic pattern and have the same means and variances and power spectra as their corresponding original time series. Secondly, DFA and sample entropy were calculated for all 1404 (39 surrogate iterations × 9 participants × 4 conditions) surrogate time series. Finally, the DFA and sample entropy from the original time series were statistically compared to the DFA and sample entropy from the surrogate data sets (see below). A significantly lower DFA or sample entropy in the original time series will reject the null hypothesis and indicate that the fluctuation in the original time series is not generated by a linearly autocorrelated Gaussian process. The results of the surrogate analyses are presented graphically in Supplementary Material 3.

### Statistical analyses

Paired Student's *t*-test was used to evaluate differences in dependent measures between PSR on the stairmill and PWS on the treadmill. To establish if statistical persistence or anti-persistence was present in the treadmill and stairmill climbing stride time, stride length, and stride speed time series, one-sample *t*-tests were applied to test if there were significant differences between the observed alpha-values and 0.5. A significantly higher observed alpha-value compared to 0.5 would indicate the present of statistical persistence and a significantly lower alpha-value would indicate statistical anti-persistence. No significant difference would indicate that the dynamics of the investigated time series would resemble uncorrelated noise. One-way ANOVA for repeated measures was performed to determine differences in dependent measures between the stairmill conditions. If the ANOVA yielded a significant result, Tukey *post-hoc* tests were applied. A one-sample *t*-test was used to establish differences in alpha-values and sample entropy between original and surrogate time series. A significance level of 0.05 was used for all the statistical tests. Statistical power and Cohen's d effect size were calculated for the *t*-tests results. Cohen's d effect size above 0.8 was considered large, between 0.5 and 0.8 moderate and below 0.5 small. In addition, statistical power at *p* = 0.05 was calculated for the ANOVA results. All statistical analyses were performed in SigmaPlot (Systat Software, Inc. 2014, version 13.0, Germany).

## Results

### Stride interval, stride length and stride speed characteristics during continuous stair-climbing and treadmill walking

The mean stride time, standard deviation and coefficient of variation of stride time during treadmill walking at PWS were significantly lower compared to continuous stair-climbing at 100%PSR (all *p* < 0.001; Table 1). The mean alpha-value was not significantly different (*p* = 0.388) but the sample entropy was significant lower in treadmill walking (*p* < 0.001; Table 1). When compared to 0.5 the alpha-value from the stride time during treadmill walking at PWS was significantly greater (*p* = 0.006) while stair-climbing at 100%PSR was not. In contrast, the alpha-value from the stride length did not significantly differ from 0.5 during treadmill walking but was significantly lower during stair-climbing (*p* < 0.001).

**Table 1:** Mean (standard error of mean) of dependent variables of right leg stride time of treadmill walking at preferred walking speed and stair-climbing at preferred stepping rate.

	**Treadmill Walking**	**Stair-climbing at PSR**	**Mean difference [confidence interval]**	***P*-value, statistical power and effect size**
Mean interval time (s)	1.08 (0.04)	2.29 (0.14)	1.21 [0.88–1.56]	< 0.001, 100%, 0.90
Standard deviation (s)	0.01 (0.003)	0.09 (0.01)	0.08 [0.04–0.09]	< 0.001, 100%, 0.82
Coefficient of variance (%)	1.30 (0.18)	3.62 (0.30)	2.32 [1.73–2.91]	< 0.001, 100%, 0.84
Alpha	0.60 (0.03)^*^	0.54 (0.07)	−0.06 [−0.11–0.24]	0.394, 12.4%, 0.20
Sample entropy	1.01 (0.11)	2.31 (0.11)	1.30 [0.94–1.66]	< 0.001, 100%, 0.89

* *Indicates a significant difference compared to 0.5*.

The mean stride length was significantly longer during treadmill walking at PWS compared to 100%PSR during stair-climbing (*p* < 0.001; Table 2). There was a significant difference in the standard deviation of stride length between treadmill walking and stair-climbing (*p* = 0.04). The stride length coefficient of variation was significantly greater during 100%PSR stair-climbing compared to PWS treadmill walking (*p* = 0.014). The mean alpha-value was significantly greater in treadmill walking compared to stair-climbing (*p* < 0.001; Table 2) but sample entropy was not significantly different. The stride length alpha-value during stairmill climbing was significantly different from 0.5.

**Table 2:** Mean (standard error of mean) of dependent variables of right leg stride length of treadmill walking at preferred walking speed and stair-climbing at preferred stepping rate.

	**Treadmill walking**	**Stair-climbing at PSR**	**Mean difference [confidence interval]**	***P*-value, statistical power and effect size**
Mean stride length (m)	1.29 (0.058)	0.66 (0.004)	−0.62 [−0.49 – −0.76]	< 0.001, 100%, 0.93
Standard deviation (m)	0.02 (0.001)	0.0155 (0.001)	0.0045 [−0.0014 – −0.0069]	0.009, 85.7%, 0.47
Coefficient of variance (%)	1.60 (0.23)	2.35 (0.15)	0.75 [0.31 – 1.19]	0.004, 93%, 0.54
Alpha	0.56 (0.03)	0.27 (0.02) ^*^	−0.29 [−0.23 – −0.35]	< 0.001, 100%, 0.87
Sample entropy	2.14 (0.03)	2.31 (0.11)	0.17 [−0.07–0.41]	0.148, 29.2%, 0.33

* *Indicates a significant difference compared to 0.5*.

The mean stride speed was significantly higher during treadmill walking at PWS compared to 100%PSR during stair-climbing (*p* < 0.001; Table 3). There was a significant difference in the standard deviation of stride speed between treadmill walking and stair-climbing (*p* < 0.001). The stride speed coefficient of variation was significantly greater during 100%PSR stair-climbing compared to PWS treadmill walking (*p* < 0.001). There were no significant differences for the alpha-value and sample entropy in the stride speed between treadmill and stairmill (Table 3). The stride speed alpha-value during treadmill walking was not significantly different from 0.5.

**Table 3:** Mean (standard error of mean) of dependent variables of right leg stride speed of treadmill walking at preferred walking speed and stair-climbing at preferred stepping rate.

	**Treadmill walking**	**Stair-climbing at PSR**	**Mean difference [confidence interval]**	***P*-value, statistical power and effect size**
Mean stride speed (m/s)	1.21 (0.08)	0.30 (0.02)	−0.91 [−0.73 – −1.11]	< 0.001, 100%, 0.93
Standard deviation (m/s)	0.015 (0.0009)	0.009 (0.0004)	−0.006 [−0.004 – −0.007]	< 0.001, 100%, 0.80
Coefficient of variance (%)	1.30 (0.16)	3.24 (0.22)	1.94 [1.66–2.23]	< 0.001, 100%, 0.86
Alpha	0.42 (0.04)	0.47 (0.06)	0.04 [−0.135–0.222]	0.591, 7.9%, 0.14
Sample entropy	2.07 (0.02)	2.12 (0.05)	0.05 [−0.07–0.18]	0.380, 13.0%, 0.22

### Influence of stepping rate on stride characteristics and variability during continuous stair-climbing

**Figure 2:**
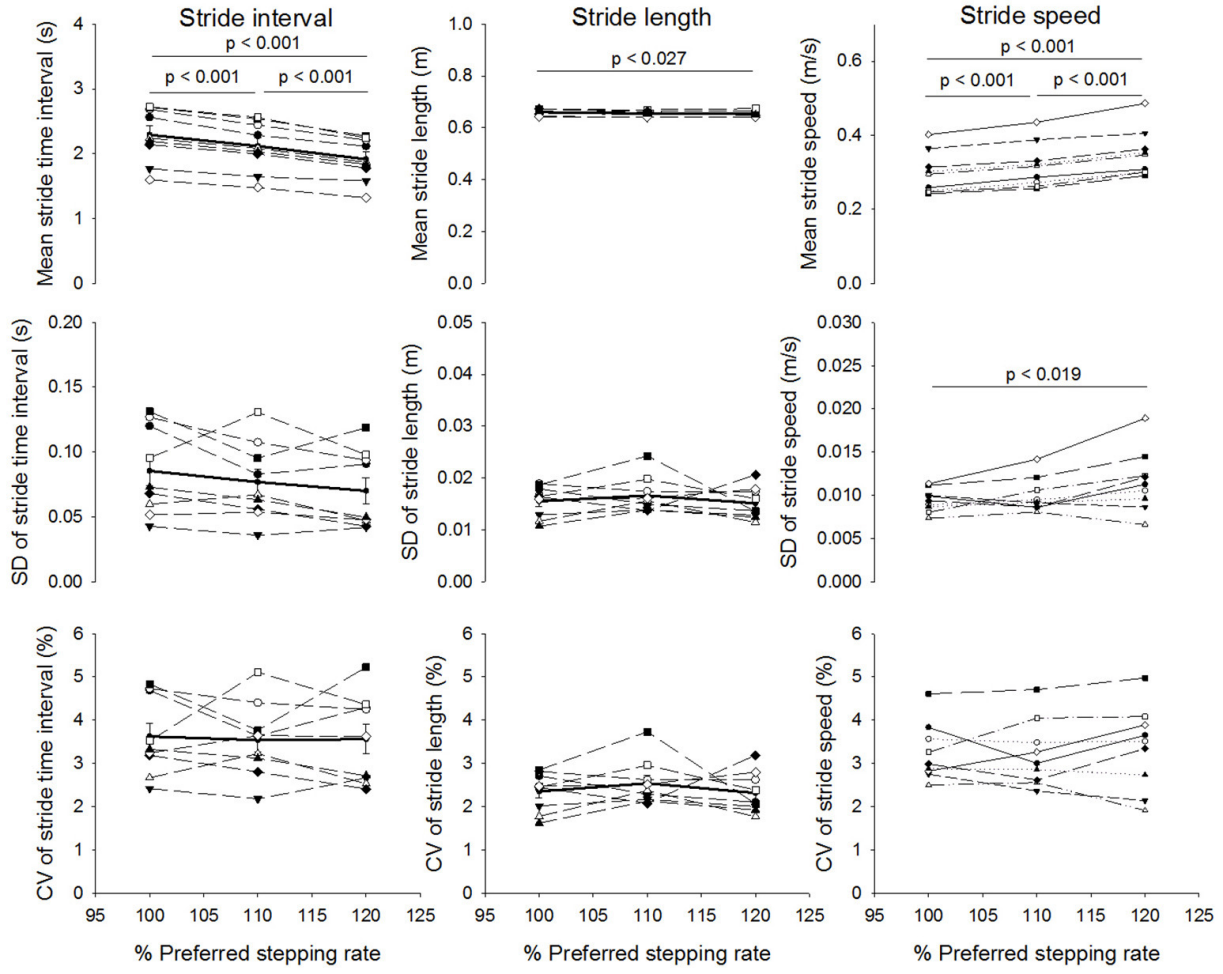
Mean ± standard error of mean (top graphs), standard deviation ± standard error of mean (middle graphs) and coefficient of variation ± standard error of mean (bottom graphs) of stride time intervals, stride length and stride speed for the three stairmill conditions based on preferred stepping rate (PSR): 100%PSR, 110%PSR and 120%PSR. Solid lines: group mean. Dash lines: individual values. *P*-values indicate between stepping rate significant differences.

**Figure 3:**
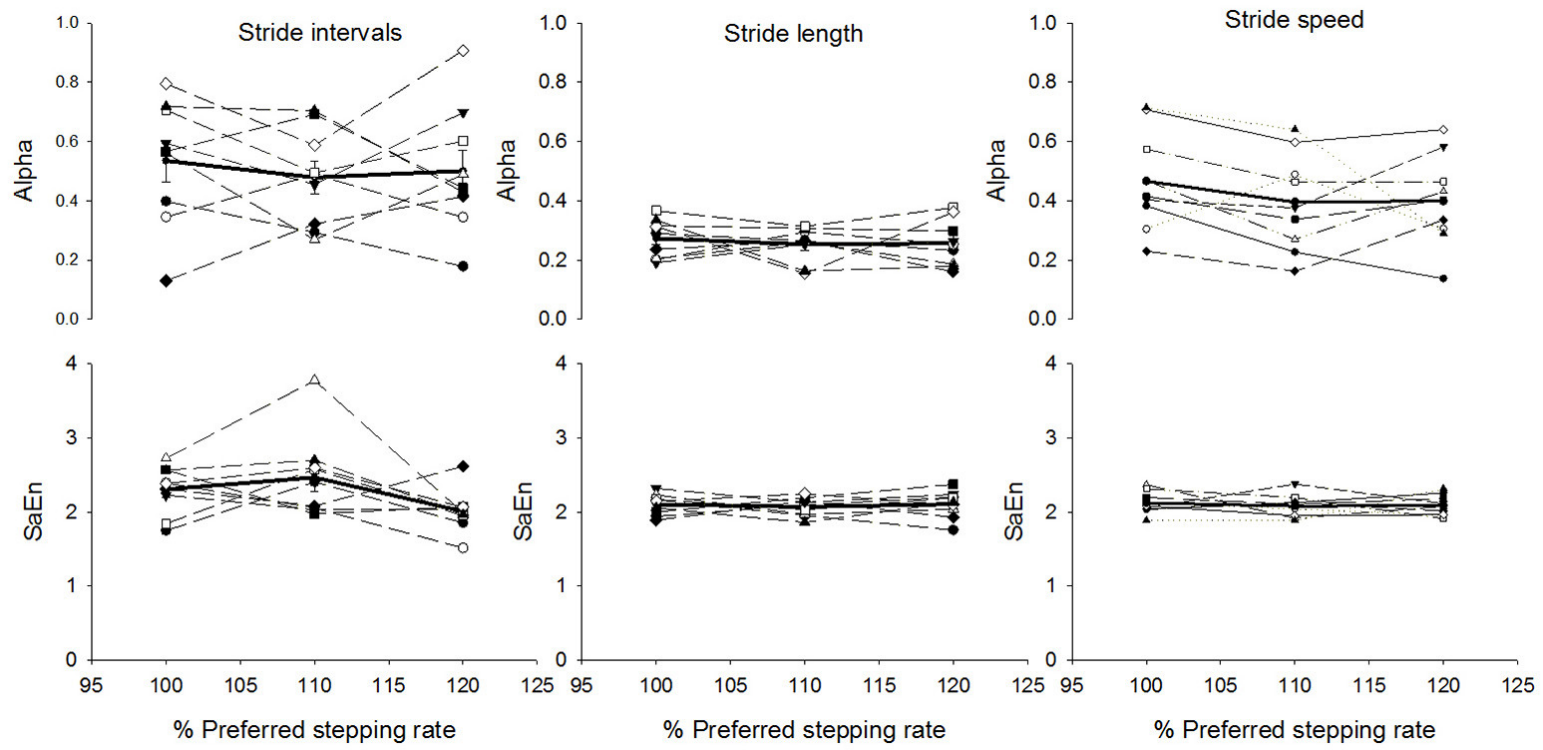
Mean ± standard error of mean of alpha-values (top graphs) and sample entropy (bottom graphs) for the stride time intervals, stride length and stride speed for the three stairmill conditions based on preferred stepping rate (PSR): 100%PSR, 110%PSR and 120%PSR. Solid lines, group mean; Dash lines, individual values.

There was a significant effect of stepping rate on the mean stride time (*p* < 0.001; statistical power at *p* = 0.05: 100%). The *post-hoc* analysis showed that mean stride time significantly decreased as the stepping rate increased and all the conditions differed significantly with each other (7.4% decrease from 100%PRS to 110%PSR and 9.6% decrease from 110%PSR to 120%PSR, *p* < 0.001; Figure 2). There was also a significant effect of stepping rate on the mean stride length (*p* = 0.024; statistical power at *p* = 0.05: 59.8%). The *post-hoc* analysis revealed a significant decrease from 100%PSR to 120%PSR (*p* = 0.023). This decrease was relatively small (0.007 m corresponding to approximately 1; Figure 2). In addition, there was a significant effect of stepping rate on the mean stride speed (*p* < 0.001; statistical power at *p* = 0.05: 100%). The *post-hoc* analysis showed that the mean stride speed increased with each increment in stepping rate (7.4% increase from 100%PSR to 110%PSR and 10.0% increase from 110%PSR to 120% PSR, *p* < 0.001; Figure 2). There was no significant effect of stepping rate of standard deviation or coefficient of variation on stride time or on stride length but the standard deviation of the stride speed significantly increased from 100%PSR to 120%PSR (*p* = 0.019; Figure 2). The alpha-values of stride time, stride length and stride speed were not affected by change in stepping rate (Figure 3). It should be noted that the differences between participants in the alpha-value and sample entropy were larger when calculated on stride time and stride speed compared to stride length.

### Surrogate analysis

When comparing the alpha-value from the original treadmill walking stride interval time series at the PWS with alpha-values from the surrogate time series 4 out of 9 participants showed significantly lower values in the original time series, 4 participants showed higher alpha values and one participant showed no difference. For the stride length time series 5 participants had significant lower alpha-value when compared to the corresponding surrogate time series and 4 participants had a significant higher alpha-value. For the stride speed time series 7 participants had significant lower alpha-value when compared to the corresponding surrogate time series and 2 had significant higher alpha-values. All participants had significantly lower sample entropy for the original treadmill walking stride interval time series at the PWS when comparing to the sample entropy from the surrogate time series. For the stride length time series 4 participants had significant lower sample entropy when compared to the corresponding surrogate time series two participants had a significantly higher alpha-value and for the remaining 3 participants there was no significant difference between the original time series and the corresponding surrogate time series. For the stride speed time series all participants had significantly lower sample entropy for the original time series compared to the corresponding surrogate time series (see Supplementary Material).

The surrogate analyses for stair-climbing revealed that alpha-values calculated on 48.1% of stride time interval time series, on 40.7% of stride length time series and on 51.9% of stride speed time series were significantly lower compared to the alpha-values from their corresponding surrogate time series. Furthermore, sample entropy calculated on 48.1% of stride time interval time series, on 37.0% of stride length time series and on 63.0% of stride speed time series were significantly lower compared to the sample entropy from their corresponding surrogate time series (see Supplementary Material).

## Discussion

Previous studies have confirmed the presence of statistical persistence in stride-to-stride fluctuations during locomotion activities like walking (Hausdorff et al., 1995) and running (Jordan et al., 2006) and that the strength of persistence was speed-dependent (Jordan et al., 2006, 2007; Jordan and Newell, 2008). In the present study, we examined if this was true for continuous stair-climbing. Results of the current study showed that statistical persistence were absent in stride time intervals and a more irregular stride pattern was observed during continuous stair-climbing compared to treadmill walking. Furthermore, the stride length during stair-climbing showed presence of anti-persistence with the same irregularity as during treadmill walking. Statistical persistence was absent in stride speed fluctuations during both stairmill climbing and treadmill walking and both conditions showed high irregularity. We also observed that increased stepping rate did not change the temporal structure of the stride-to-stride fluctuations in stride time, stride length and stride speed. Thus, the results of the present study were more in line with those studies indicating a lack of speed dependency during walking and running (Dingwell and Cusumano, 2010; Terrier and Deriaz, 2012; Lindsay et al., 2014).

### Walking vs. continuous stair-climbing

In the current study, an alpha-value of 0.60 for stride time intervals at PWS for treadmill was consistent with previous studies that suggested the presence of statistical persistence (Hausdorff et al., 1995; Jordan et al., 2007). This statistical persistency suggests that the characteristics of the stepping pattern in terms of stride interval remain consistent throughout the entire time series and that the stride time of a stride at a later time-point is dependent on a stride time of a stride at an earlier time-point. The current study resulted in an alpha-value of 0.54 for continuous stair-climbing, suggesting an absence of such statistical persistence. Such a value also indicates the presence of white noise and an absence of scaling and fractal behavior (Hausdorff et al., 1995). It should be noted that large variations were observed between participants in the present study. Thus, both persistence and anti-persistence in the stride time intervals were observed within the group of participants. This could indicate that large individual differences in the control strategies existed. However, methodological bias due to the short data series cannot be excluded (see further discussion below). Significantly higher sample entropy during stair-climbing compared to treadmill walking supported this observation. The surrogate analysis revealed that in less than half of the stride time data series the fluctuations could be generated by a linearly autocorrelated Gaussian process. Taken together, these observations indicate that the fluctuations present in stride time during stair-climbing resemble a random and uncorrelated pattern.

The alpha-value of stride length during treadmill walking was 0.56 which is lower than what has been previously reported (Jordan et al., 2007; Dingwell and Cusumano, 2010). The observed mean alpha-value did not statistically differ from 0.5 indicating that the stride length fluctuations were uncorrelated. The relatively high irregularity quantified by sample entropy supports this observation of randomness in the stride length fluctuations. The alpha-value of stride length during stair-climbing indicates presence of statistical anti-persistence with an equally high irregularity as during treadmill walking. In contrast to the stride time intervals, the inter-participant variation in alpha-values for stride length was relative small. Thus, a general pattern of anti-persistence in stride length time series was observed across participants.

The low alpha-value of 0.42 (not statistically different from 0.5) calculated on the stride speed fluctuations during treadmill walking, is well in line with previous observations (Dingwell and Cusumano, 2010; Dingwell et al., 2010) and is supported by the high irregularity. The stride speed fluctuations during stairmill climbing resembled that observed during treadmill walking. This could indicate that although different motor strategies could be used to control stride time intervals and stride length during the two walking conditions, the applied motor control strategies results in indistinguishable stride speed fluctuations (Dingwell and Cusumano, 2010). Similar to the stride time intervals, large inter-participant variations in alpha-values of stride speed time series was observed. Thus, no clear general pattern could be observed across subjects.

In addition to significant differences in sample entropy of the stride time and alpha-value in stride length between treadmill and stair-climbing, significantly greater absolute and relative variability (standard variation and coefficient of variation) were observed in stair-climbing. These significant differences could have been due to the challenges associated with continuous stair-climbing. Participants were more comfortable to walk on the treadmill at a greater stepping rate (smaller stride interval). Continuous stair-climbing is a more challenging task than treadmill walking and probably requires more involvement from central nervous system resources.

It is possible that during continuous stair-climbing, each step is considered by the central nervous system as a new problem to solve and minimal feedback from the previous step(s) is required. This would indicate a more adaptive control where each step is based on the constraints placed on the individual who is not accustomed to performing continuous stair-climbing. Mechanically, while someone walking on a treadmill is free to choose from a range of combinations of stride rate and length to produce a given speed, someone walking on a stairmill can primarily vary their stride rate to produce a given speed. Hence, continuous stair-climbing places constraints on the individual to produce sufficient vertical and forward motion of the foot to successfully accomplish the task. This problem may require a new solution at each step thereby producing a greater amount of variability, absence of statistical persistence and reduced regularity during continuous stair-climbing. In addition, Hausdorff et al. (1996) showed that walking in response to a metronome results in weaker statistical persistence in stride patterns. It is possible that the continuous stair-climbing task using the stairmill device where the steps are in set heights and distances, places similar constraints on an individual and thus, result in uncorrelated stride time fluctuations. This task-specificity of the temporal structure of variability was also highlighted by Jordan et al. (2006).

The continuous rhythmical re-occurrence of steps during stairmill climbing could induce a constraint on the participants similar to that of visual cues during treadmill walking (Terrier, 2016). Terrier observed that statistical persistence in stride length during treadmill walking changed to anti-persistence when the participants were required to adjust their heel strikes to rectangular “stepping stones” displaced on the treadmill belt (Terrier, 2016). Furthermore, increased variability quantified by coefficient of variation of stride lengths was observed (Terrier, 2016). This is well in line with the results of the present study, where both statistical anti-persistence and a higher coefficient of variation in stride length was observed during stairmill climbing compared to treadmill walking.

### The effect of speed on continuous stair-climbing

In several studies, Jordan and colleagues observed that the strength of statistical persistence was weakest at preferred walking and running speeds compared to 20 and 10% slower and faster speeds (Jordan et al., 2006, 2007). This U-shaped pattern at different speeds could suggest that individuals feel most comfortable and ready to adapt while walking at their PWS resulting in a lower strength of persistence. This observation could be explained by a reduction in the adaptability at slower and faster speeds causing an increased dependency on the feedback of initial steps on the later steps (Jordan et al., 2006, 2007). In contrast to these observations, Terrier and Deriaz (2012) and Dingwell and Cusumano (2010) observed that the statistical persistence did not change at velocities 30 and 20% below and above PWS. The results of the current study for continuous stair-climbing showed that the fluctuations in stride time and stride speed were uncorrelated and did not change significantly with increase in stepping rate. Additionally, the relatively high irregularity was not affected by changes in the stepping rate. This would imply that no correlation between consecutive strides exists. Interestingly, a constant statistical anti-persistence in stride length with relatively small inter-participant variation for all three stepping frequencies was observed across different stepping rates. This indicates that a short stride was likely to be followed by a longer stride and vice versa.

Compared to treadmill walking at different speeds, continuous stair-climbing produced greater mean stride time, mean stride length, coefficient of variation of stride time and length, but lower alpha-values of stride time and stride length (Jordan et al., 2007). This could imply that performing a more challenging task like continuous stair-climbing might place an additional demand on the neuro-muscular system resulting in higher variability in the stride characteristics, a more random stride pattern and reduced adaptability.

One way to manipulate gait adaptability is by providing additional feedback. Indeed, our previous work has shown that the strength of persistence can be affected during walking by manipulating the optic flow speed of Virtual Reality environments (Katsavelis et al., 2010). It is possible that the strength of persistence during stairmill climbing may also be affected by providing additional feedback. This could be a goal for future research. Furthermore, PWS has been modeled as a pendulum and has been strongly correlated with limb length and the inertia of the limb. It also coincides with the most energetically efficient walking speed (Ralston, 1958; Zarrugh et al., 1974). Whether, this is true in case of the PSR during stair-climbing needs to be further investigated.

## Study limitations

When applying nonlinear analysis (e.g., DFA and sample entropy) on physiological time series, the length of the time series in question is critical to the reliability of the results (Eke et al., 2000, 2002; Delignieres et al., 2005; Yentes et al., 2013). Investigating the effect of data length on the precision of the alpha-value calculated on fractional Gaussian noise and fractional Brownian noise, Eke et al. (2002) observed that reliability was compromised when using data length below 4,000 data points. However, Delignieres et al. (2005) concluded that when applying DFA to physiological time series approximately 500 data points provides acceptable results. When investigating the application of sample entropy on stride interval time series, Yentes et al. (2013) suggested time series length above 200 data point for reliable results. Although the number of strides (*N* = 65 for stairmill climbing and *N* = 167 for treadmill walking) is low compared to these recommendations and what previous studies have used for similar investigation during walking (Hausdorff et al., 1996; Jordan et al., 2007; Yentes et al., 2013), the additional analysis presented in the Supplementary Material showed that this did not affect the reliability of the results. The results of the Supplementary Material indicate that DFA and sample entropy calculated on mathematical signals (white, pink, and brown noise) were more sensitive to change in data length compared to the biological signals (stride time, stride length, and stride speed). Especially, the group mean of both DFA and sample entropy calculated on stride time, stride length, and stride speed did not change significantly with changes in data length (from 50 to 165 strides). However, the individual results (Figure 3) shows large inter-participant variation of the alpha-values calculated on stride intervals and stride speed but small inter-subject variation on stride length. As suggested above, this could indicate inter-participant differences in motor control strategies. However, it cannot be excluded that these differences were caused by the methodological limitation of the short data series. Although, longer data series would be preferable, the challenging nature of the continuous stair-climbing task might limit the physical capacity of the participants to allow for longer data collection. It should be noted that the observed increase in stride length from 100% PSR to 120% PSR during stairmill climbing is very small (7 mm–1%) and could lie within the accuracy range of the motion capture system. This increase in stride length could also originate from a slight different foot position on each step at higher stepping rate. The current study is limited by not including DFA and sample entropy for continuous stair-climbing at speeds slower than the PSR. Another limitation of the present study was the relatively low sampling frequency. The present study based stride characteristics on kinematic data recorded at 60 Hz. This means that there was a stride time detection accuracyof 0.0167 s/frame. With a mean stride time during treadmill of 1.08 s, this accuracy corresponds to approximately 1.5% of the variable.

## Conclusion

In spite of large inter-participant variations, the group average indicated that statistical persistence was absent in stride interval during continuous stair-climbing at PSR. In contrast, stride length fluctuations were characterized by statistical anti-persistence which was consistent across all participants. In addition, stride time fluctuations were more irregular during stair-climbing compared to treadmill walking. Furthermore, increase in stepping rate did not change the nonlinear characteristics or the amount of variability in the stride pattern.

## Ethics statement

This study was carried out in accordance with the recommendations of the Institutional Review Board of the University of Nebraska Medical Center with written informed consent from all subjects. All subjects gave written informed consent in accordance with the Declaration of Helsinki. The protocol was approved by the Institutional Review Board of the University of Nebraska Medical Center.

## Author contributions

SV, JR, MM, and NS designed and performed the experiment. PR, SV, JR, MM, and NS conducted the analyses and interpreted the results. PR, SV, MM, and NS drafted the manuscript and all authors approved the final version. All experiments were performed at University of Nebraska at Omaha.

### Conflict of interest statement

The authors declare that the research was conducted in the absence of any commercial or financial relationships that could be construed as a potential conflict of interest.
