# Supplementary material for: Dynamics of Stride Interval Characteristics during Continuous Stairmill Climbing
Source: Front Physiol. 2017 Aug 23;8:609. doi: 10.3389/fphys.2017.00609 (PMC5572333; doi:10.3389/fphys.2017.00609)
Supplement: Supplementary file 1 [file Presentation1.pdf]

SUPPLEMENTARY MATERIAL

Title:

Dynamics of stride interval characteristics during continuous stairmill climbing

Authors:

Peter C. Raffalt[1,2], Srikant Vallabhajosula[3], Jessica J. Renz[4], Mukul Mukherjee[4] and Nicholas Stergiou[4,5]

Affiliations:

[1] Julius Wolff Institute for Biomechanics and Musculoskeletal Regeneration, Charité – Universitätsmedizin Berlin, Germany.

[2] Department of Biomedical Sciences, University of Copenhagen, Copenhagen, Denmark.

[3] Department of Physical Therapy Education, School of Health Sciences, Elon University, Elon, NC, United States

[4] Department of Biomechanics, College of Education, University of Nebraska at Omaha, Omaha, NE, United States

[5] Department of Environmental Agricultural and Occupational Health, College of Public Health, University of Nebraska Medical Center, Omaha, NE, United States

Corresponding Author:

Nicholas Stergiou, PhD

Department of Biomechanics and Center for Research in Human Movement Variability

University of Nebraska at Omaha

6160 University Drive

Omaha, NE 68182-0860, USA.

Email: nstergiou@unomaha.edu

Phone: 402-554-3247

Supplementary material 1

The purpose of this supplementary material 1 was to investigate the effect of different box sizes on the scaling exponent obtained with detrended fluctuation analysis (DFA).

Twenty examples of three different mathematical signals of 2500 data points with different characteristics (periodic, chaotic, and random) were created using the *Colored noise generator* function in Matlab (MathWorks R2011b). These signals included a brown noise signal (power spectrum of $1/f^2$) which was considered to be periodic, a pink noise signal (power spectrum of 1/f) which was considered to be chaotic, and a white Gaussian noise signal (constant power spectral) which was considered to be random. These time series were cut to include either 65 data points (very short), 165 data points (short), or 2000 data points (long). The scaling exponent of white noise signal of long data series lengths (>2000 data points) can be expected to be close to 0.5, of pink noise close to 1.0, and of brown noise close to 1.5. In this case the use of different box sizes should have very limited effect on the precision of the DFA. To verify this claim, the present investigation included data series of 2000 data points. Furthermore, to investigate the effect of box size of relative short data series for the DFA, the present investigation included very short and short data lengths.

DFA was performed with four different box sizes of [2,N], [4,N], [8,N] and [16,N] for each time series. In order to best fit a curve to the linear part of the log N-log F(N) plots, two different scaling regions were chosen, 1:14 for the very short and short time series and 20:50 for the long time series (figure S1.1-S1.3). One-sample t-tests were applied to investigate if the alpha values from each of the four box sizes significantly differed from 0.5, 1.0, and 1.5, respectively. To account for multiple statistical comparisons, level of significance was set to 0.001.

Across the three short signals, box size of [2,N] provided a scaling exponent closest to the expected (figure S1.1-S1.3) for the very short and short time series. For the long time series, choice of box size had less effect on the precision of the scaling exponent. However, large box sizes appeared to provide scaling exponent with greater difference to the expected value (figure S1.1-S1.3). When using the longest data series there was a general tendency to a smaller deviation from the theoretical value and a smaller standard deviation compared to the shorter data series. Thus, the present supplementary material supports the used box size in the main study.

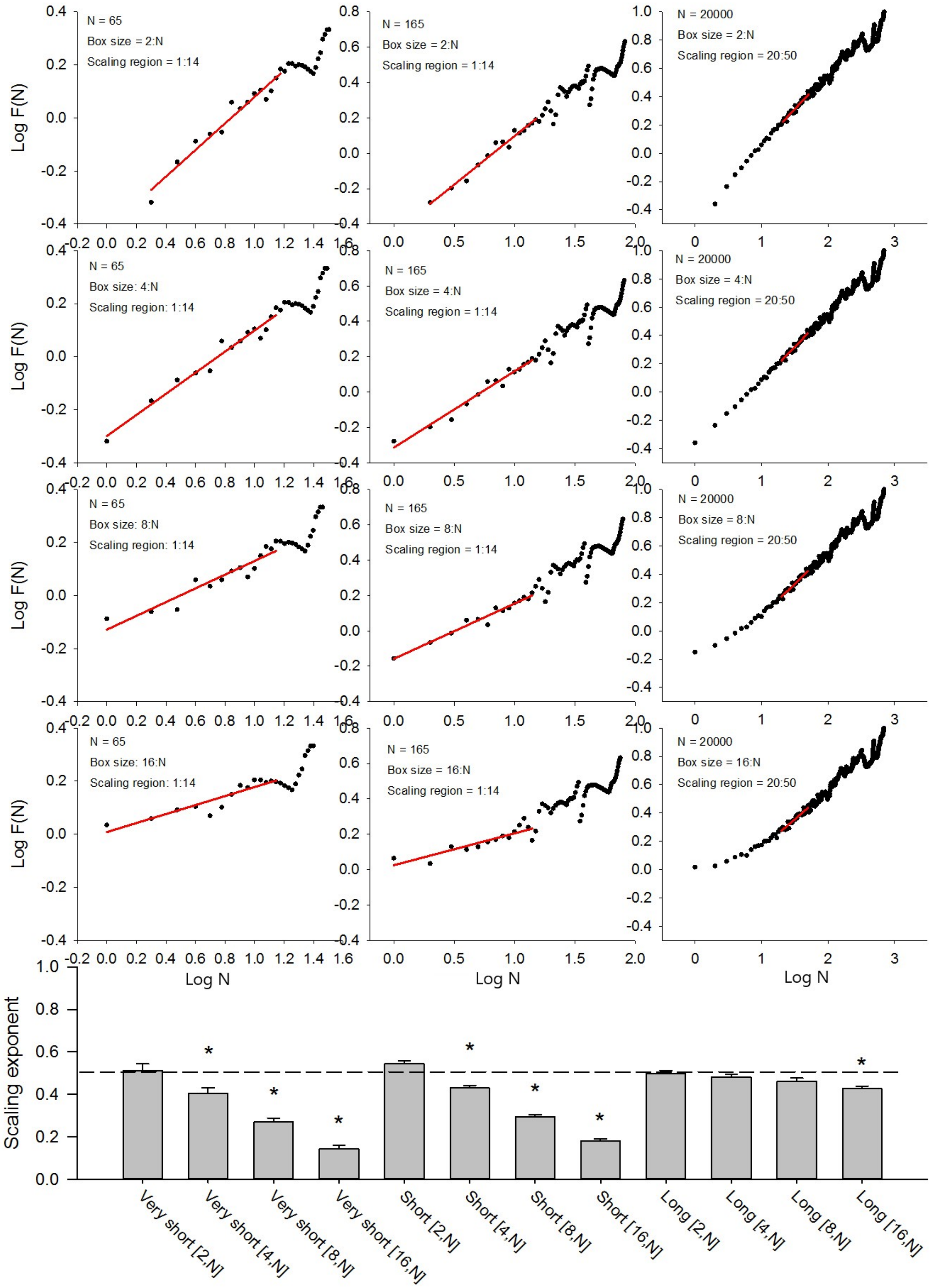

Figure S1.1: Representative log N–log F(N) plots for white noise signals with different time series length (left graphs: N=65, middle graphs: N=165, right graphs N=20000) different box sizes (first row: box size=2:N, second row: box size=4:N, third row: box size=8:N, fourth row: box=16:N) , and different scaling regions (left and middle colon: scaling region=1:14, right colon: scaling region 20:50). Bottom graph: mean and standard deviation of scaling exponents for twenty iterations of white noise when using different time series length, box sizes, and scaling region. Significant difference ($p<0.001$) between the average scaling exponent and 0.5 is indicated by asterisks.

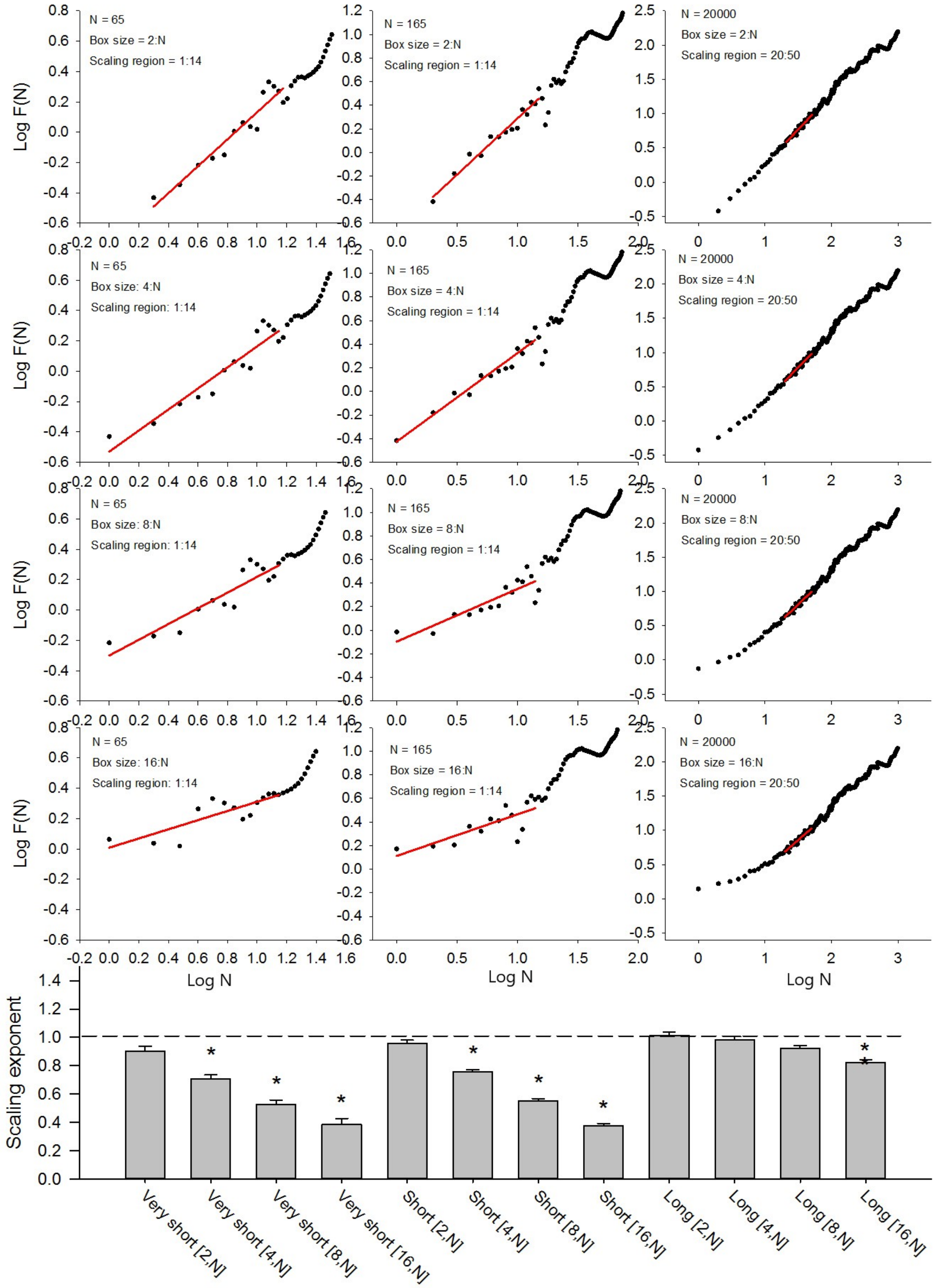

Figure S1.2: Representative log N–log F(N) plots for pink noise signals with different time series length (left graphs: N=65, middle graphs: N=165, right graphs N=20000) different box sizes (first row: box size=2:N, second row: box size=4:N, third row: box size=8:N, fourth row: box=16:N) , and different scaling regions (left and middle colon: scaling region=1:14, right colon: scaling region 20:50). Bottom graph: mean and standard deviation of scaling exponents for twenty iterations of pink noise when using different time series length, box sizes, and scaling region. Significant difference ($p<0.001$) between the average scaling exponent and 1.0 is indicated by asterisks.

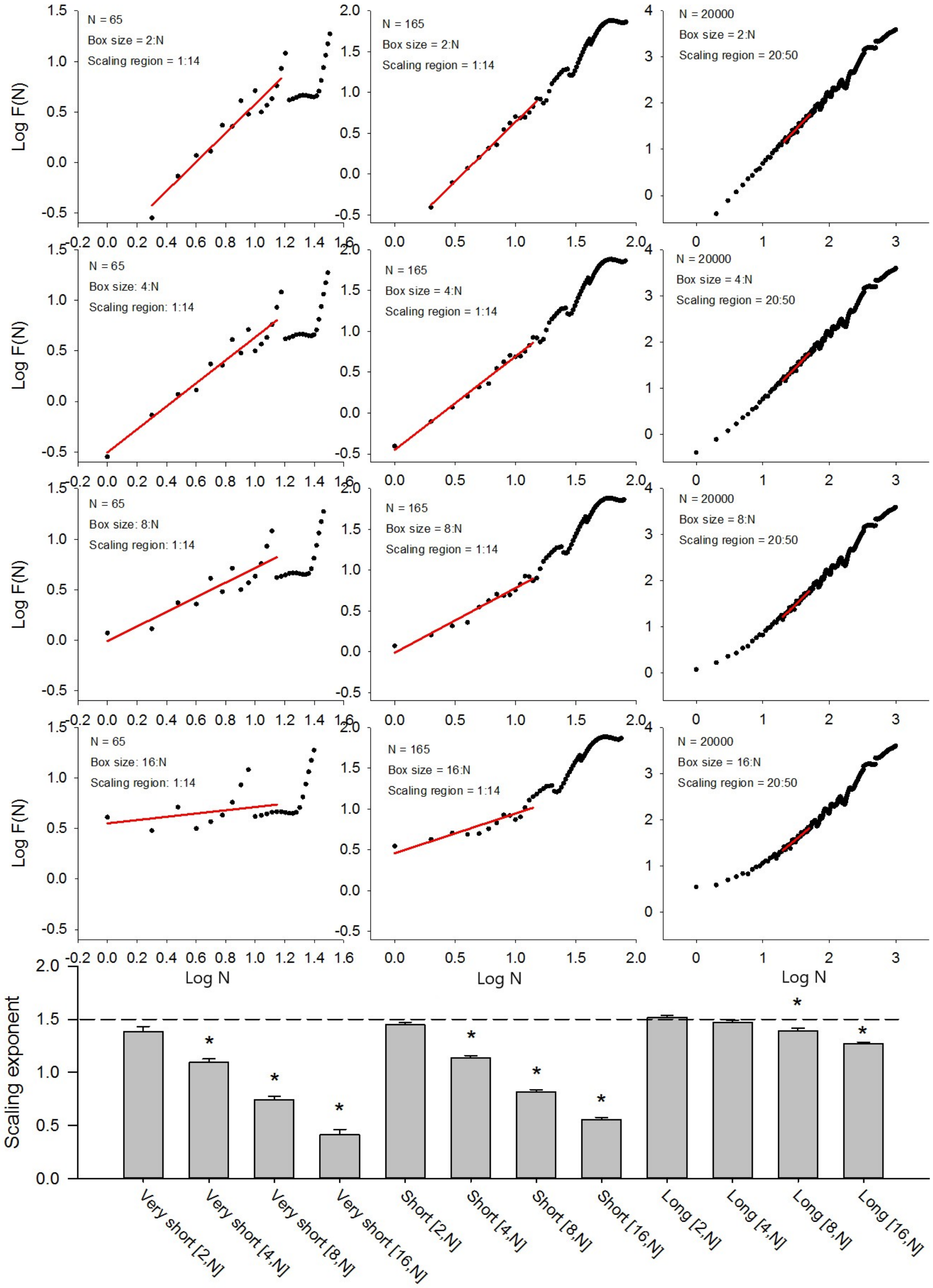

Figure S1.3: Representative log N–log F(N) plots for pink noise signals with different time series length (left graphs: N=65, middle graphs: N=165, right graphs N=20000) different box sizes (first row: box size=2:N, second row: box size=4:N, third row: box size=8:N, fourth row: box=16:N), and different scaling regions (left and middle colon: scaling region=1:14, right colon: scaling region 20:50). Bottom graph: mean and standard deviation of scaling exponents for twenty iterations of brown noise when using different time series length, box sizes, and scaling region. Significant difference ($p<0.001$) between the average scaling exponent and 1.5 is indicated by asterisks.

Supplementary material 2

The purpose of this supplementary material 2 was to validate that the number of included strides did not affect the reliability of the DFA and SaEn. The twenty time series of three different mathematical signals described in supplementary material 1 were also used in this investigation. Additionally, the stride time, stride length, and stride speed time series during the walking trial from each of the nine subjects were included. Each time series (both mathematical and biological) were resized into nine different time series with a length ranging from 50 to 165 strides (figure S2.1). DFA and SaEn were calculated from each of the 540 theoretical time series (3 mathematical signals x 20 iteration x 9 different time series lengths) and each of the 81 biological time series (9 subject x 9 different time series lengths). A one-way ANOVA for repeated measures with a level of significance at 0.05 was applied to test the effect of the length of time series. In case of a significant effect, a Holm Sidak post hoc test was applied. In addition, the standard deviation across all iterations of the mathematical signals (figure S2.1) and across all subjects for each of the 9 different time series length was plotted against the number of data points (figure S2.2).

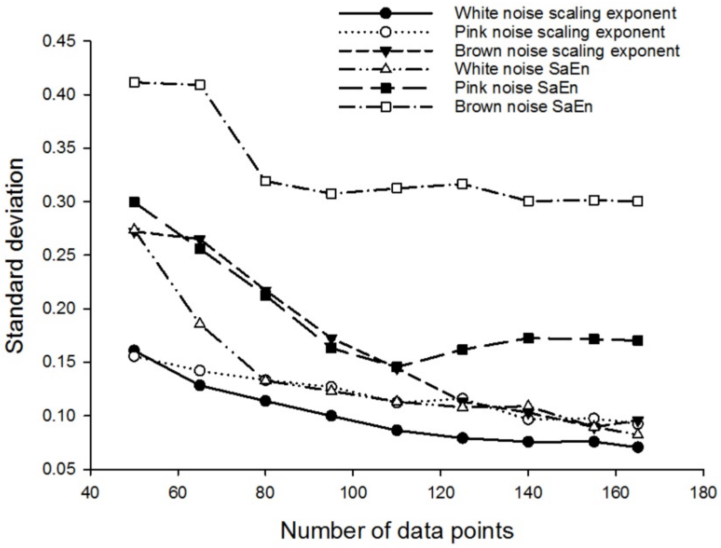

Figure S2.1: Standard deviation of the scaling exponent and the SaEn calculated on the white noise, pink noise, and brown noise time series as a function of different time series lengths.

The standard deviation of the scaling exponent and SaEn calculated on different noise signals (figure S2.2) did change with increase the number of data points. While the standard deviation of the scaling exponent of all three signals and the SaEn of the white noise signal decreased gradually with increase in included data points, the SaEn of both the pink and brown noise signal appeared to level off and remain relative constant with data lengths above 80 and 95 data points, respectively. The standard deviation of the different stride variables (figure S2.2) did not appear to change substantially when 95 or more data points were used.

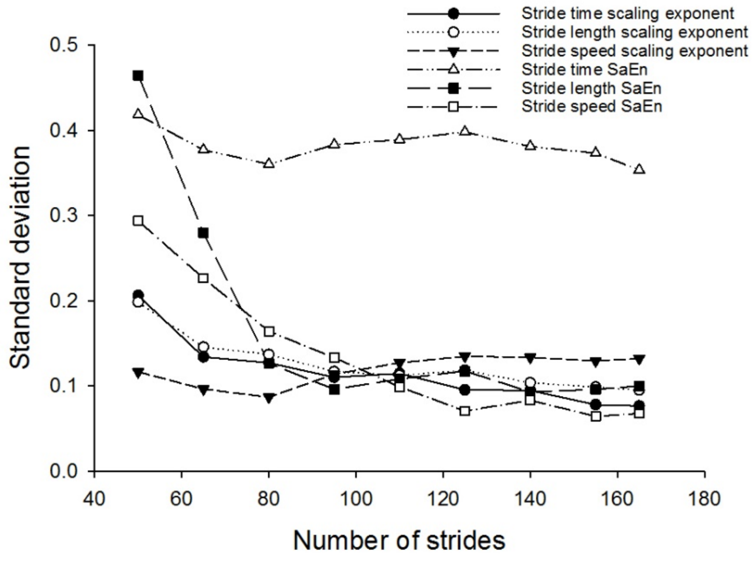

Figure S2.2: Standard deviation of alpha values and SaEn calculated on stride time and stride length as a function of different time series lengths.

When comparing the average scaling exponent and SaEn for the twenty iterations of the three signals at different data series length (figure S2.3), no significant effect was observed for the white and pink noise signals. A significant effect of data series length was observed for brown noise ($p=0.012$). The post hoc test revealed that the average scaling exponent at a data length of 125 and 165 data points was significantly higher compared to the scaling exponent at data length 50 ($p<0.039$ and $p<0.030$, respectively). For the average SaEn, there was significant effect of data length for all three noise signals ($p\leq0.002$ in all cases). The post hoc test showed that the average SaEn for the white noise signal at data length 65 was significantly higher compared to that of data length 165. For the pink noise signal, the SaEn for data lengths of 50 to 95 data points was in all cases significantly higher than the corresponding values for data lengths of 110 to 165 data points. For the brown noise signal, SaEn was significantly higher for data length 50 compared to the six longest data lengths, SaEn for data length 65 was significantly higher compared to the four longest data lengths, and SaEn for data length 80 was significantly higher compared to the two longest data lengths (figure S2.3). There was no significant effect of the data length of the stride variable time series on DFA and SaEn (figure S2.4).

While the presented results on mathematical signals indicate a significant effect of data length on both reliability and precision of the variable outcome, this does not seem to be the case with biological data such as the stride time, stride length, and stride speed used in the present study. While figure S2.2 does indicate that the between subject variance increased when few data points were used, the results presented in figure S2.4 indicate that the groups average was less affected by changes within the range of data point used in the present study.

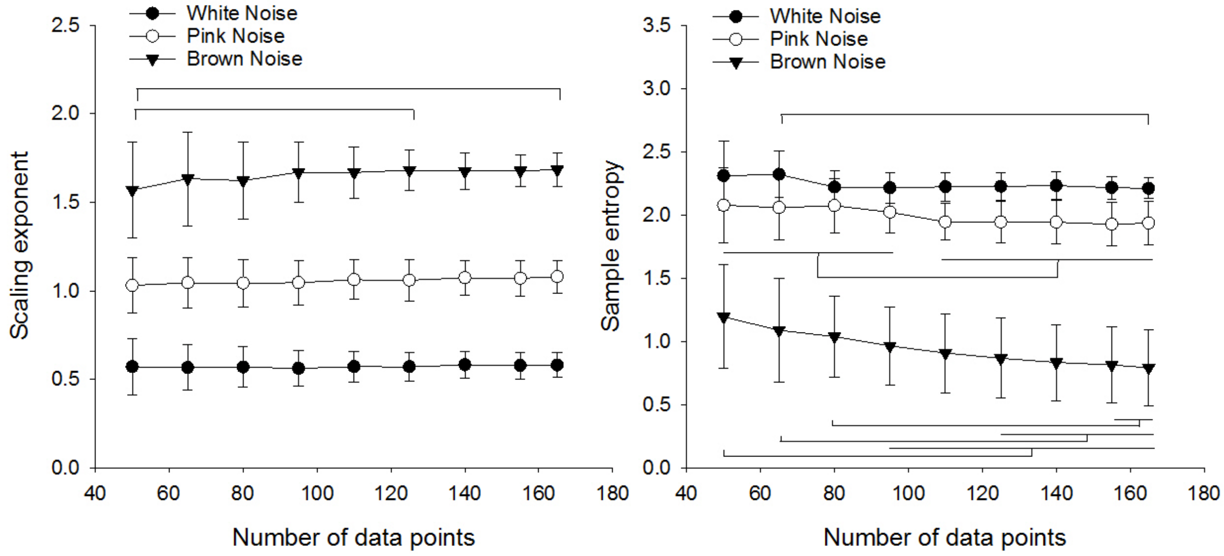

Figure S2.3: DFA (left graph) and sample entropy (right graph) calculated on white noise, brown noise, and pink noise time series of different length. Note: horizontal lines indicate significant differences.

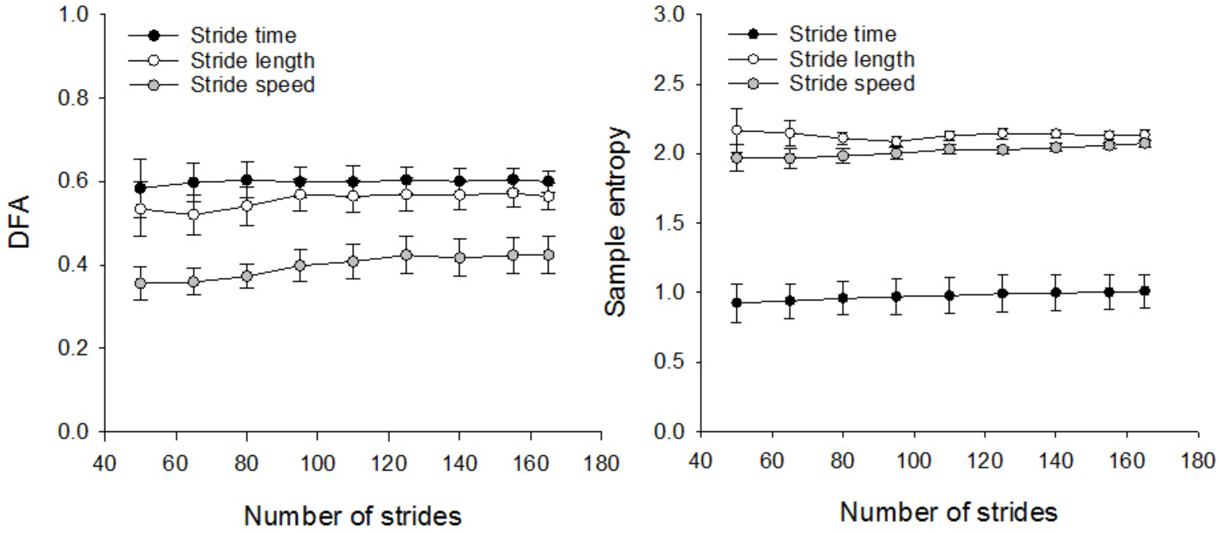

Figure S2.4: DFA (left graph) and sample entropy (right graph) calculated on stride time, stride length and stride speed time series of different length.

Based on the presented results, it can be concluded that the reliability and precision of DFA and SaEn was only limited affected by the data series length when biological data was used. This was not the case for mathematically generate signals.

Supplementary material 3

This supplementary material presents the surrogate analysis results. In each figure the dependent variable (alpha-values or sample entropy on stride time interval, stride length or stride speed) is plotted as a star for each subject. In addition, the dependent variable is calculated on 39 surrogate time series and plotted as box plots with 95% confidence intervals. The results of the statistical analysis are presented in the main study.

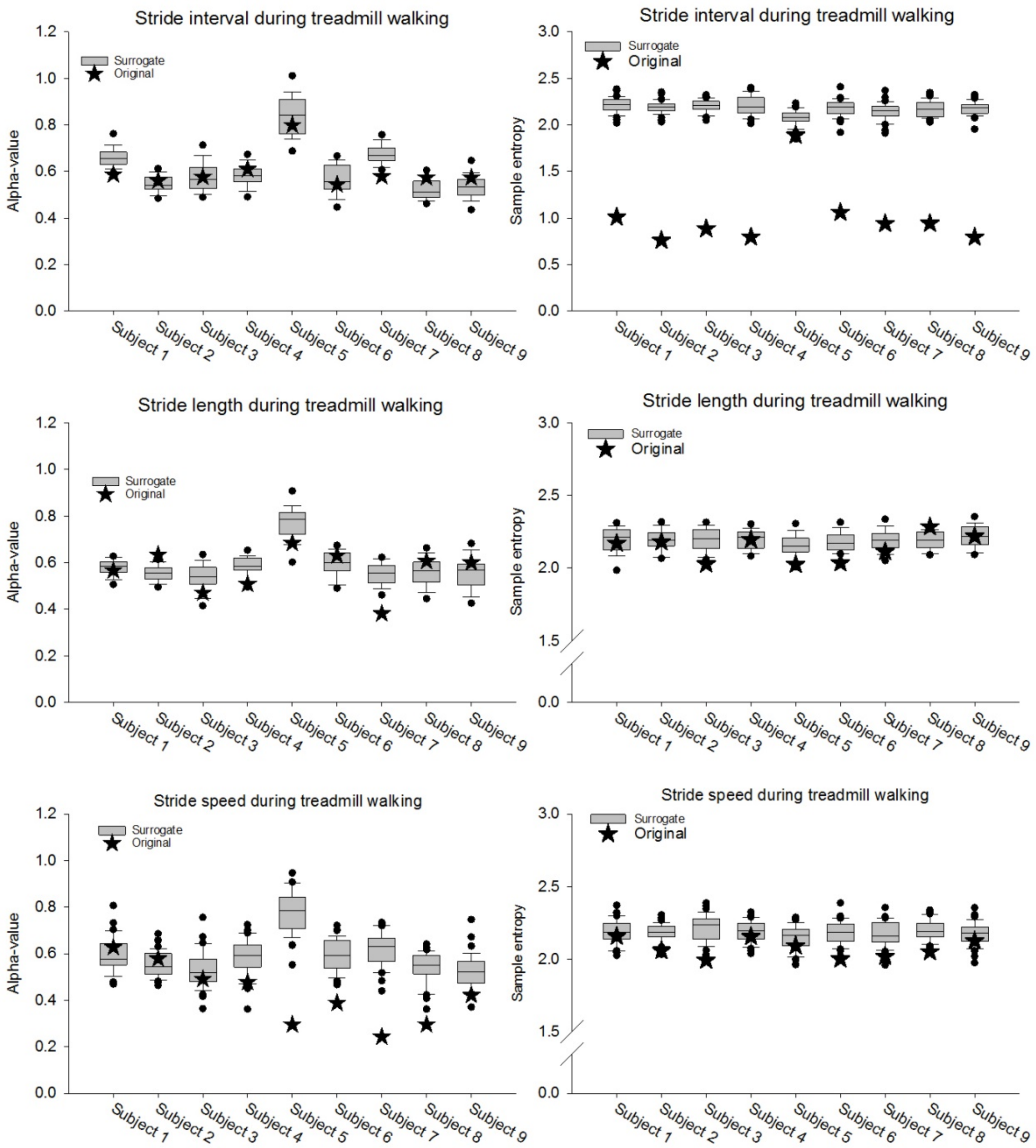

Figure S3.1: Alpha-value (left graphs) and sample entropy (right graphs) calculated on surrogate time series (boxplot) and original time series (star) for stride time intervals (top graphs), stride length (middle graphs) and stride speed (bottom graphs) for each subject during treadmill walking at preferred walking speed.

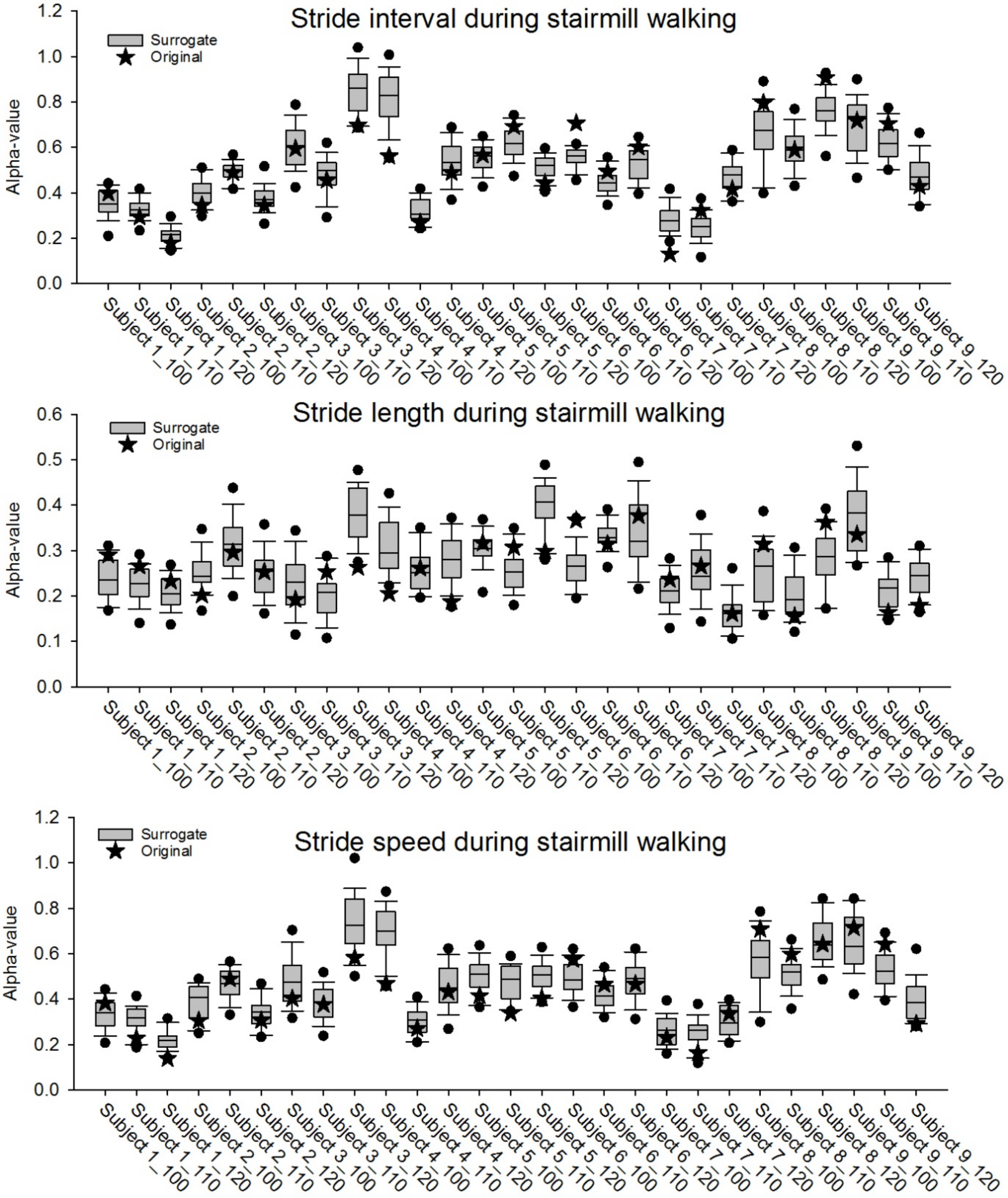

Figure S3.2: Alpha-value calculated on surrogate time series (boxplot) and original time series (star) for stride time intervals (top graphs), stride length (middle graphs) and stride speed (bottom graphs) for each subject during stairmill walking at 100%, 110% and 120% of preferred stepping rate.

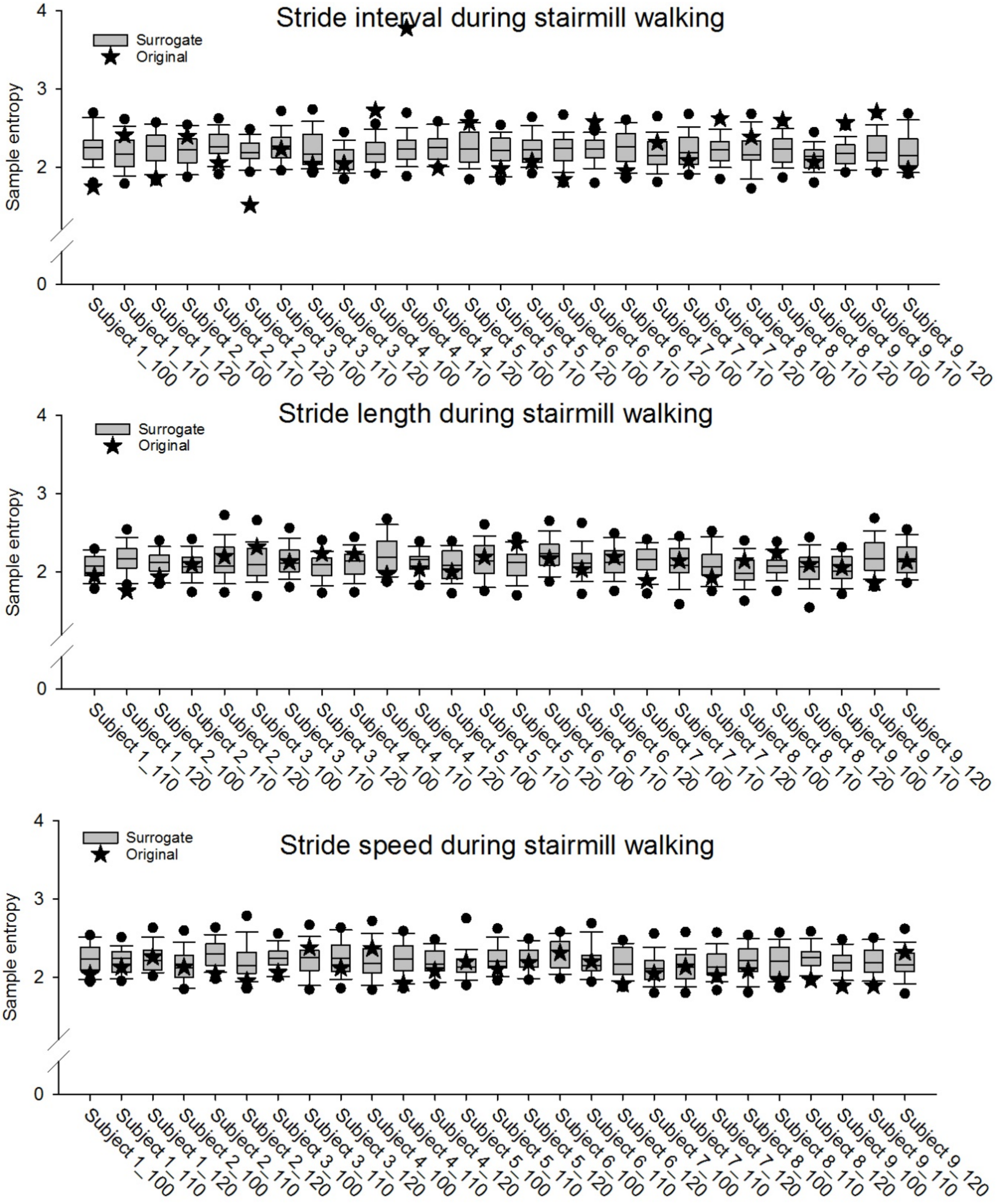

Figure S3.3: Sample entropy calculated on surrogate time series (boxplot) and original time series (star) for stride time intervals (top graphs), stride length (middle graphs) and stride speed (bottom graphs) for each subject during stairmill walking at 100%, 110% and 120% of preferred stepping rate.
